# Using social media for assessment purposes: Practices and future directions

**DOI:** 10.3389/fpsyg.2022.1075818

**Published:** 2023-01-18

**Authors:** Dennis Alonzo, Cherry Zin Oo, Wendi Wijarwadi, Caitlin Hannigan

**Affiliations:** ^1^School of Education, University of New South Wales, Kensington, NSW, Australia; ^2^Department of Educational Pyschology, Yangon University of Education (YUOE), Yangon, Myanmar; ^3^Department of Educational Management, Faculty of Teaching and Educational Science, Syarif Hidayatullah State Islamic University Jakarta, South Tangerang, Indonesia

**Keywords:** social media, assessment, schools, learning, teaching

## Abstract

The use of social media across the world is rapidly increasing, and schools are advancing its use for learning, teaching, and assessment activities. Despite growing evidence for their accessibility and affordances for educational purposes, very little attention has been paid to their use in assessment. Using the Preferred Reporting Items for Systematic Reviews and Meta-analyses (PRISMA), this paper is an initial step to explore how social media have been used and reported in the literature, and describe some key challenges. A total of 167 articles were initially accessed from three databases, but only 17 were relevant after applying the exclusion criteria. Results show that the most dominant social media used in assessment are Facebook and Twitter. Also, the assessment practices are limited to sending and discussing assessment tasks, following up on progress, giving feedback, and engaging in self and peer assessment. Key issues include the trustworthiness of the assessment process and outputs, limited features of social media platforms, technical support, time commitment between teachers and students, and intersections of social and academic engagements. We discuss the implications of these findings with the critical gaps in the theorisation of using social media for assessment purposes.

## 1. Introduction

Social media are used for various purposes, including communicating, sharing information, creating content, dating, and many others (Alalwan, 2022). Statista (2021) predicts that social media users will rise to 4.41 billion people worldwide by 2025. In educational contexts, schools are also advancing their use for learning, teaching, and assessment activities. Social media have become highly interactive platforms where students can share and discuss their ideas and information (Ashraf et al., 2021; Mahmud et al., 2022). Using social media in learning and teaching, including facilitating interactions between teachers and students and among students, is not geographically limited or time-bound (Akgül and Uymaz, 2022). Thus, providing a mechanism for continuous transfer and co-construction of knowledge together. In addition, social media use facilitates synchronous and asynchronous interactions with many additional features, including links and resource sharing, and voice messaging (Ashraf et al., 2021). Moreover, the learning platform in social media facilitates parents to monitor their children’s progress as they can access their work. Hence, it creates a more significant interaction for students, teachers, and parents/careers. In addition, by engaging in social media learning, students also create an opportunity to interact with people worldwide (Espinoza-Celi and Pintado, 2020). Other people become resources that can provide critical insights to their learning. As such, social media have become the most favored alternative platforms for educational systems that cannot subscribe to learning management systems (Al-Rahmi et al., 2022).

While this evidence highlights the use of social media for learning and teaching, there are only a few reports on how social media are used for assessment purposes. It has been shown that social media can facilitate the provision of immediate feedback (e.g., Annamalai and Jaganathan, 2017; Nuray, 2019). Teachers could respond as soon as students uploaded their work (Liu and Ko, 2020). Through this immediate feedback, students could monitor their learning progress and how they are meeting the learning outcomes (Espinoza-Celi and Pintado, 2020). In addition, the use of social media can facilitate peer assessment. Students can comment on each other’s posts, identifying their strengths and providing suggestions to further improve their peer’s work. This process increases students’ motivation and confidence in learning (Vikneswaran and Krish, 2016; Lai et al., 2020). There is also evidence that social media have been used to administer pre- and post-tests to measure students’ learning gains (e.g., Huang et al., 2014; Yuk and Yunus, 2021). Collectively, these few research articles provide evidence for social media use as alternative platforms for implementing assessment. However, it is quite limited with only few assessment strategies used. It is also unclear from the literature the intersections between assessment and social media use. Given that assessment plays a central role in improving learning and teaching (Assessment Reform Group, 2002; Black and Wiliam, 2018), and social media are gaining prominence as alternative platforms in learning and teaching, it is worthwhile exploring the use of social media for assessment purposes. Thus, we provide an initial step to scope how social media have been used in assessing students and the issues associated with their use in primary and secondary school contexts. We aim to identify critical gaps in the literature and provide future research directions. The following research questions guide this paper:

1. What are the main characteristics (e.g., study location, school level, and research design) of included studies reporting social media used in assessment?
2. What and how have social media been used for assessment?
3. What are the issues with using social media in assessment?

## 2. Literature review

We provide a brief review of the literature to provide evidence of using social media in learning, teaching and assessment activities.

### 2.1. Assessment in education

Assessment plays a central role in ensuring effectiveness of learning and teaching (Black and Wiliam, 2018). Theoretical and empirical evidence support the claim that assessment is collectively the most important intervention in the classroom with the highest effect size on increasing student outcomes (Hattie, 2009). However, despite this widely shared view, assessment processes and practices have long been debated in educational research (Bloom et al., 1971; Ramaprasad, 1983; Baird et al., 2017), with dichotomies emerging between summative (SA) and formative assessments (FA). The common understanding is that FA is routinely carried out to gather data to inform pedagogy (Bennett, 2011) and SA is used to evaluate whether learning has occurred (Lau, 2016). To address this issue, the Assessment Reform Group (2002) proposed the concept of Assessment *for* Learning (A*f*L) and defined it as “a process of seeking and interpreting evidence to identify where learners are, where they need to go, and how best to go there” (Assessment Reform Group, 2002, p. 2). This definition encompasses all assessment strategies used in the classrooms, including FA and SA, which the results are used to inform learning and teaching activities. Building on this definition, Davison (2007) offered a continuum of assessment practices from in-class contingent FA, planned formative assessment, mock SA, to the most formal SA, including high stake testing and international examinations whose results are used to support individual students. More recently, Black and Wiliam (2018) explicitly argued that that the dichotomy between FA and SA becomes irrelevant when assessments are conceptualized within a broader pedagogical model.

To optimize the impact of assessment on student learning, regardless of types, they should be an integral part of learning and teaching, and the results are used to identify learning needs of, and support needed by individual students. The design, implementation, and participation of students in the assessment process are critical for its effectiveness. There is a growing consensus in education that teaching, learning and assessing should aim to foster and develop students to actively engage, participate, contribute to, reflect on and evaluate their learning approaches and outcomes (Hannigan et al., 2022). Moreover, the social interactions and the dialogic nature of assessment (Ruiz-Primo, 2011), particularly eliciting and giving feedback, as conceptualized from a Vygotskian perspective, calls for student agency in the learning space and the necessity of knowledge exchange, regardless of the type of assessment in use. As such, exploring how social media can be used as alternative platforms for assessment purposes, in an ever changing and increasingly dynamic educational landscape is important as educators seek to engage students in increasingly innovative and responsive ways, and create opportunities for students to demonstrate their learning.

### 2.2. Online assessment

The number of online resources available for learning and teaching has increased because of the advancement of information and communication technology (Özden et al., 2004). Subsequently, the use of online learning has increased dramatically, creating additional opportunities for interaction among students, students and teachers, and teachers themselves (Singh and Thurman, 2019). In comparison to traditional classroom settings, learning practices have changed as a result of the growth of online learning, allowing higher interactions among students and opportunities for students to adopt a more flexible approach (Robles and Braathen, 2002).

Moving classes from a traditional classroom setting to an online setting means that learning practices has fundamentally been shifted, including assessment techniques (Robles and Braathen, 2002). For example, the development of e-learning influences the broader opportunities for innovation in assessment beyond the limitation of the traditional paper-based test (Scalise and Gifford, 2006). In addition, within an online course, learning activities and assessment are very closely connected since teachers needs to carefully articulate the desired learning objectives and how those objectives are measured through an online assessment approach (Sewell et al., 2010). Moreover, the practice of online assessment depends greatly on teachers’ competencies since it requires technical skills in scoring and providing real-time feedback for students (Olufisoye and Ola, 2013). The practice of online assessment is fundamentally dominated by summative assessment, measuring the overall learning achievement of students (Guangul et al., 2020). Herein, most online assessment platforms are built for one-way interaction where students are presented with assessment tasks and respond to it accordingly (Schultz and Callahan, 2022). These platforms do not allow for more meaningful assessment practices that mimic the classroom environment where teachers can implement assessment as an integral part of learning and teaching processes (Black and Wiliam, 2018).

Thus, social media can be viewed as an alternative platform for administering educational assessment. It has some distinctive features when compared with online assessment platforms. Social media also offers the opportunity for students to receive timely feedback from fellow classmates (Kio, 2015). Social media is primarily formative in nature and offers a more dialogic and interactive feedback as well as prompt responses from teachers, whereas online assessments are primarily delivering a one-way approach (teacher to student). In addition, the barriers of delayed responses from the assessment are diminished in social media platforms since student can ask questions any time, and the teacher can provide an immediate response to students’ question.

### 2.3. Social media in education

Social media have become an integral platform for learning and teaching that helps students share ideas, bridge communication gaps, and browse information (Mahmud et al., 2022). Giroux and Moreau (2022) demonstrate teachers’ use of social media to engage students both in structured and unstructured learning. In structured learning, they include watching YouTube videos in the classroom and using the content for further discussion. Unstructured learning activities using social media include finding relevant learning content as informal learning. Herein, evidence indicates that students use social media to communicate and fulfill their educational needs (Salih and Elsaid, 2018). For example, students use social media to improve their writing skills by posting their draft and receiving feedback from their peers and teacher (Haidari et al., 2020).

Different types of social media have been used in teaching, including Twitter, WhatsApp, Instagram, YouTube, and Snapchat (Alenezi and Brinthaupt, 2022; Noori et al., 2022). Research indicates that students have specific preference for the type of social media they use to support their learning (Ma et al., 2021). Facebook, including Messenger, is the widely preferred communication tool among students (Maulina Geelan et al., 2021), and students use it to communicate, share ideas, and discuss their assignments (Donlan, 2014). On the other hand, even though Instagram has not received much attention for learning and teaching, some teachers use it to engage students in learning activities (Handayani, 2015). In the study of Mahmud et al. (2022), classroom teachers asked students to post Instagram stories about their reading activities, including photos of their favorite books and a short book review. Their study reveals that this activity increased student motivation to read and learn. WhatsApp has also been used as a collaborative tool for sharing learning materials with students (Mangundu, 2022), and interacting with students beyond the classroom setting (Maulina Geelan et al., 2021).

The collaboration and engagement among students and teachers *via* social media have enhanced opportunities to share ideas and discuss them (Pujiati et al., 2019; Ansari and Khan, 2020; Alenezi and Brinthaupt, 2022). Teachers use social media to assign group posts in the course (Lai et al., 2021), where students then create groups on social media sites to engage in discussion, regardless of their physical presence. Students in the study of Donlan (2014) reported on the benefits of said social media usage in that they “can constantly communicate with each other like send links out, send photos, all the research we find. So, we all get it and all share it rather than texting someone or trying to email so we are all included in the same thing (p. 583).”

### 2.4. The use of social media for assessment purposes

Previous research highlighted using social media for assessment purposes to support students’ learning. The use of social media in assessment can cultivate increasingly creative and enjoyable learning opportunities for students. Teachers use social media as a platform for students to discuss and submit their outputs (Mahmud et al., 2022). In addition, teachers use social media sites to assess student outcomes (Alabdulkareem, 2015), particularly by providing timely feedback (Giroux and Moreau, 2022). The interactivity of social media serves as a platform for students to clarify feedback (Alfahadi, 2017). Students can interact with their teachers to seek clarifications on the content of feedback and ask for suggestions on how to act on them. Lai et al. (2021) used social media to assign various tasks in the course and utilize various opportunities for students to engage in classroom activities. They have found out that the use of social media for learning and assessment purposes provide students greater sense of participation and ownership.

Specific social media have been used in assessment. For example, Facebook is used to enhance students’ vocabulary. The interactivity, provision of immediate feedback, and availability of online resources facilitate students’ learning and use of new words (Mukhlif and Challob, 2021). In addition, Facebook is used to improve students’ reflective writing (Annamalai and Jaganathan, 2017). Students engage in peer assessment providing feedback to their peers. Twitter is used to develop students’ writing skills by learning sophisticated vocabulary, expressions, idioms, and grammar structures (Espinoza-Celi and Pintado, 2020). Edmodo is used to develop the writing competence of primary school students at the pre-writing and drafting stage of writing (Yuk and Yunus, 2021).

While there is evidence for the use of social media for assessment purposes, there are reported issues related to its uptake and teachers’ and students’ beliefs and actual use. For example, in the study of Donlan (2014), Facebook was used for communication and assessment. However, the uptake is relatively low due to the perceived use of Facebook for personal space, and not for assessment purposes. There was a mismatch between the potential use of Facebook and students’ attitude. This is corroborated by the study of Alabdulkareem (2015) that found that teachers and students are willing to use social media in assessment, but the actual practice is relatively low. Teachers and students still hold a strong belief that social media is for socialization platform only.

### 2.5. Impacts of using social media in learning and teaching

Using social media in learning and teaching has demonstrated several advantages. First, using social media creates stronger relationships between students and teachers, which students can benefit from through collaboration (Ashraf et al., 2021; Akgül and Uymaz, 2022). By participating collaboratively on social media, students can create highly interactive environments where they can create and exchange ideas and interact with their teachers (Ashraf et al., 2021). In the study of Akgül and Uymaz (2022), research postulates that collaboration is one of the most significant variables impacting the educational use of Facebook. Second, students have higher trust in collaboration with their peers toward enhancing involvement and participation (Alalwan, 2022) because they can communicate freely and easily (Mahmud et al., 2022). Higher trust in collaboration results in ‘increased involvement, participation, the reason for using social media, usefulness, and accessibility of the use’ (Alalwan, 2022, p. 9786). Last, embedding social media use in classroom pedagogy can enhance students’ learning performance by collaborating and engaging in learning activities that transform students from passive receivers to active learners (Alenezi and Brinthaupt, 2022).

## 3. Materials and methods

To answer the research questions, we conducted a literature review, including a search and analysis of the initial data collated from databases relevant to the study, then refined the selection for data synthesis according to the Preferred Reporting Items for Systematic Reviews and Meta-Analyses (PRISMA) guidelines (Moher et al., 2009).

### 3.1. Data sources and literature search

An initial search of the literature was conducted through ProQuest (ERIC and Education), and Scopus. We first extracted all studies related to using social media for assessment in schools, published up to May 2022. We did not set a lower boundary for the year of publication to include and review important earlier research. The combination of keywords*, social media, Facebook, Twitter, TikTok, Instagram, YouTube, WhatsApp, Snapchat, Pinterest, Reddit, LinkedIn, student, formative assessment, summative assessment, feedback, self-assessment, peer assessment, assessment for learning, assessment of learning, assessment as learning, questioning, classroom assessment, teacher assessment, high school, junior, senior, secondary, primary,* and *elementary, K-12,* were used to identify the papers in each database. We included the 10 social media with the highest subscriber to expound our literature search. The detailed search strategy syntax used for each database can be seen in Table 1. Articles were included in this review if they were published in peer-reviewed journals in English. There were no restrictions regarding the design of studies: quantitative, qualitative, or mixed-methods.

**Table 1:** Search strategy syntax.

Database	Syntax	Number of Articles
Web of Science	TS = [(“social media” OR Facebook OR Twitter OR Tiktok OR Instragram OR Youtube OR WhatsApp OR Snapchat OR Pinterest OR Reddit Or LinkedIn) AND (student) AND (“formative assessment” OR “summative assessment” OR “feedback” OR “self-assessment” or “peer assessment” OR “assessment for learning” OR “assessment of learning” OR “assessment as learning” OR “questioning” OR “classroom assessment” OR “teacher assessment”) AND (“high school” OR junior OR senior OR secondary OR primary OR elementary OR “K-12”)]	52
Scopus	TITLE-ABS-KEY [(“social media” OR Facebook OR Twitter OR Tiktok OR Instragram OR Youtube OR WhatsApp OR Snapchat OR Pinterest OR Reddit Or LinkedIn) AND (student) AND (“formative assessment” OR “summative assessment” OR “feedback” OR “self-assessment” or “peer assessment” OR “assessment for learning” OR “assessment of learning” OR “assessment as learning” OR “questioning” OR “classroom assessment” OR “teacher assessment”) AND (“high school” OR junior OR senior OR secondary OR primary OR elementary OR “K-12”)]	40
ProQuest	noft (“social media” OR Facebook OR Twitter OR Tiktok OR Instragram OR Youtube OR WhatsApp OR Snapchat OR Pinterest OR Reddit Or LinkedIn) AND noft (student) AND noft (“formative assessment” OR “summative assessment” OR “feedback” OR “self-assessment” or “peer assessment” OR “assessment for learning” OR “assessment of learning” OR “assessment as learning” OR “questioning” OR “classroom assessment” OR “teacher assessment”) AND [noft (“high school”) OR noft (junior) OR noft (senior) OR noft (secondary) OR noft (primary) OR noft (elementary) OR noft (“K-12”)]	121

### 3.2. Study selection

**Figure 1:**
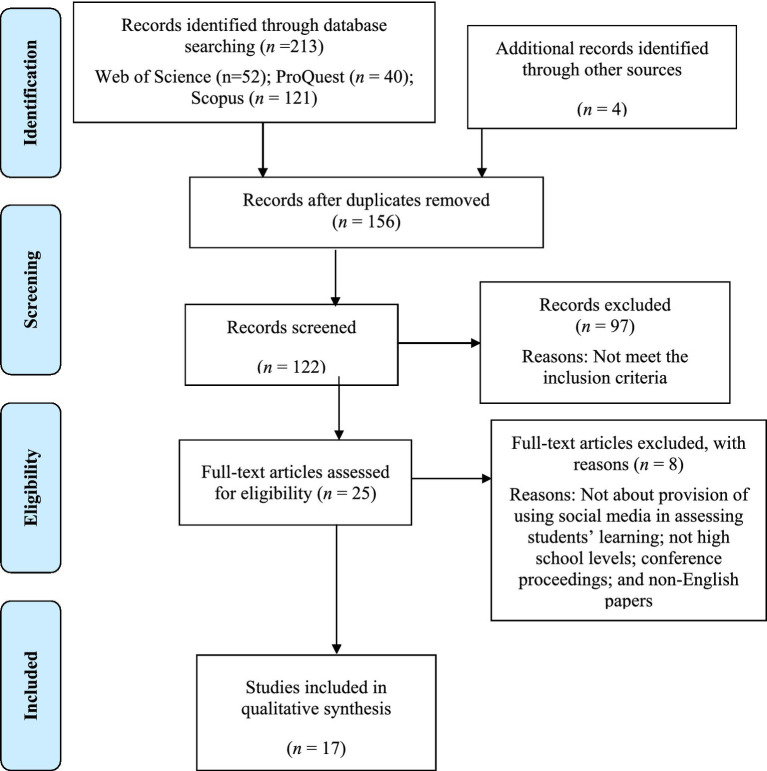
Study selection flow diagram.

The literature search based on the inclusion/exclusion criteria identified a total of 167 articles (see Figure 1). After removing the duplicates, the title and abstract of the articles were reviewed if they met the criteria. The inclusion criteria of our review were: (1) topics relating to social media in assessing students’ learning; (2) school contexts of primary or secondary school settings (excluding studies focusing on adult learning, university, and vocational education); (3) peer-reviewed journal articles; (4) use of English language; and (5) access to full-text. The title and abstract of the articles that did not clearly articulate these criteria were excluded. After applying these criteria, 24 articles remained for full-text review.

The 24 full-text articles were downloaded and carefully reviewed by the first, second, and third authors to determine the relevance of the studies for our review purpose (i.e., selecting studies on social media in assessing students’ learning). At the final stage, only 17 articles were found to be relevant to the aim of our paper. We synthesized them to answer our research questions (see Figure 1).

### 3.3. Approach to analysis and synthesis

We used the three stages of thematic synthesis for systematic reviews highlighted in Thomas and Harden (2008). These three stages are (a) line-by-line text coding; (b) identifying specific indicators; and (c) generating theoretical dimensions. The first author developed descriptive and analytical themes and were checked by the second and third. To ensure rigor and consistency of coding, the second and third authors coded the same two articles simultaneously and then compared their coding afterward. The first author confirmed the codes before moving on to the next two articles. Any inconsistencies were discussed to reach a consensus. This process was undertaken for three iterations until a higher degree of consistency was achieved. The interrater reliability (88.89%) was established by calculating the percent agreement between these two authors. The remaining articles were coded by the second and third authors while frequently negotiating on emerging inconsistencies when they arrived. The first author then checked the final coding.

## 4. Results

In this section, the results of the thematic analysis of the synthesis literature are presented to answer the research questions.

### 4.1. Characteristics of included articles

This section presents the context of social media assessment used in the 17 reviewed articles to identify the research contexts, study designs, theoretical framework, and study purposes. Descriptive analysis answers Research Question 1: *What are the main characteristics (study location, school level, research design, and types of social media used) of included studies reporting social media used in assessment?*

#### 4.1.1. Contexts of the studies

As shown in Table 2, studies on using assessment for assessment purposes were undertaken in Malaysia (*n* = 3), Taiwan (*n* = 2), China (*n* = 2), Brazil (*n* = 2), the United States, Australia, Indonesia, Ecuador, Hongkong, Iraq, Thailand, and Turkey. Asia dominated the geographical distribution with 10 out of 15 investigations conducted in Asian nations.

**Table 2:** Summary of the articles included in the review.

Authors	Country	Design	Theoretical approaches	Purposes	School level
Mukhlif and Challob (2021)	Iraq	Quasi experimental	Computer-Mediated Communication,	Comparing the use of social media and the traditional instruction in learning vocabulary	High School & Mixed
			Social Constructivism (Vygotsky)		
Yuk and Yunus (2021)	Malaysia	Quasi Experimental	Not mentioned	The use of social media in improving writing skill	High School & Mixed
Liu and Ko (2020)	Taiwan	Quasi experimental	Design Thinking: brainstorming, lateral thinking and critical judgment	The use of social media in improving students’ learning	Primary
Espinoza-Celi and Pintado (2020)	Ecuador	Survey and Field Research	Computer Assisted Learning (CALL)	The use of social media in improving writing skill	High School & Mixed
Yu et al. (2020)	China	Descriptive study	Self-Regulated Learning and Social Learning Theory of Bandura	The use of social media in improving self-regulated learning	Primary School
Lai et al. (2020)	Hongkong	Survey	Social Learning and Social Interaction	The use of social media in analyzing students’ learning	Primary school
Nuray (2019)	Turkey	semi-structured interviews	Not mentioned	The use of social media in analyzing students’ learning	High School & Mixed
Annamalai and Jaganathan (2017)	Malaysia	Case study	Reflection	The use of social media in improving writing skill	High School & Mixed
De Barros et al. (2017)	Brazil	Survey and Intervention	Complexity Thinking and	The use of social media in improving students’ learning	High School & Mixed
			Blended Learning		
Bezerra et al. (2021)	Brazil	Qualitative	Not mentioned	To analyze the creative feedback in the mathematics field	High school& Mixed
Wuttisela et al. (2016)	Thailand	Intervention	Not mentioned	The use of social media in improving students’ learning	Primary school
Vikneswaran and Krish (2016)	Malaysia	semi-structured interviews	Not mentioned	The use of social media in improving students’ writing	Primary School
Kio (2015)	China	reflective journal and survey	Feedback	The use of social media in improving students’ learning through a connected feedback model	High School & Mixed
Huang et al. (2014)	Taiwan	Quasi Experimental	Nature of Science (NOS)	The use of social media in improving students’ science learning	High School & Mixed
Casey (2013)	Australia	Action research	Not mentioned	Comparing the use of social media and the traditional instruction	High School & Mixed
Miller and Olthouse (2013)	United States	Comparative Study	Bloom’s Taxonomy	Comparing the use of social media and the traditional instruction in writing	High School & Mixed
Herwin et al. (2021)	Indonesia	Transcendental Phenomonology	Not mentioned	Analyzing the learning organization strategies during the COVID-19 pandemic	Primary School

In terms of school level, most of the research was conducted in secondary school settings. This may indicate that students’ social media literacy influences the selection of school settings (e.g., Kio, 2015; Mukhlif and Challob, 2021). Regarding research design, two strategies have dominated the study: survey studies (*n* = 4) and Quasi-Experimental (*n* = 4). The use of surveys in those studies was accompanied by another approach: field study, intervention, and reflective journal. In contrast, the quasi-experimental design adopts a similar technique consisting of a pre-test at the beginning of the study, an intervention by the researcher in one experiment group, and a post-test at the conclusion of the study to assess students’ comprehension of the supplied topics or courses.

#### 4.1.2. Purpose of the study

Four major themes in the study’s purposes emerged: social media influences on learning improvement, social media influences on learning activities, social media influences on learning assessment through feedback, and the instructional design comparison between learning through social media use and traditional method use. Although three of these themes are not directly about assessment purposes, they highlight the use of social media for assessment purposes. Hence, they provide important insights to achieve the aim of our paper.

##### 4.1.2.1. Social media and student learning improvement

Six of the 17 articles explore the influence of social media use in improving students’ language learning (e.g., Espinoza-Celi and Pintado, 2020). Social media in these papers were used as a medium to practice and improve writing skills (e.g., Miller and Olthouse, 2013; Yuk and Yunus, 2021). Teachers and students use Twitter to analyze their writing improvement (Espinoza-Celi and Pintado, 2020), while Facebook is used to investigate students’ development in writing reflection in action (Annamalai and Jaganathan, 2017). Other social media, including Peer Modo in Edmodo (Yuk and Yunus, 2021) is used to evaluate the content quality of students’ writing skills. On the other hand, StoryBird.com and KidsBlog (Miller and Olthouse, 2013) are used to examine the critical thinking skills of gifted students.

Apart from its focus on improving writing skills, other papers focus on the distinctive influences of social media use in learning. A study by Mukhlif and Challob (2021) examines the influence of Facebook in improving students’ vocabulary knowledge compared to traditional teaching for secondary school students in Iraq. In addition, a study by Yu et al. (2020) investigates some factors that influence social networking in an online self-regulated learning activity through ZhiLiao, a commercial Chinese online learning resource platform for junior high school graduates. It primarily focuses on understanding how social media benefits students’ self-regulated learning.

##### 4.1.2.2. Social media and learning activities

Besides evaluating the improvement of students’ outcomes, six articles focus more on examining the learning process. Those articles investigate how social media influences the learning dynamic. For example, a study by Vikneswaran and Krish (2016) explores what motivates English as Second Language user students to write in English on Facebook. In addition, some research examines the use of different functions of social media, such as online schoolwork sharing (Lai et al., 2020), social media-integrated teaching (Liu and Ko, 2020), and synchronous and asynchronous students’ online discussion (Huang et al., 2014). These studies highlight the critical role of social media in ensuring learning and assessment activities.

##### 4.1.2.3. Social media and learning assessment through feedback

Three articles explicitly investigated how social media contributes to students’ development by providing peer feedback. A study by Wuttisela et al. (2016) addresses the use of social media in improving students’ learning by comparing feedback provided by the students and the teachers. It strives to analyze students’ reflections as evaluators of the science projects using the D4L + P program compared to the reflections of teachers and experts in Project Based Learning. A study by Kio (2015) identifies the influence of students’ feedback on learning activities. It investigates how social media enhance students’ learning experiences through a connected feedback model provided by social media. A study by Miller and Olthouse (2013) analyzed the differences between gifted children’s offline and online feedback in a writing workshop. These three articles show that social media can be used as a learning instrument to develop students’ feedback literacy.

##### 4.1.2.4. Social media and traditional instructional design

Two articles highlight the comparison between the use of social media and traditional instructional design (Miller and Olthouse, 2013; Mukhlif and Challob, 2021). This comparison consists of two aspects: examining the effectiveness of social media use and traditional instruction use in improving students’ vocabulary knowledge (Mukhlif and Challob, 2021) and identifying the critical thinking of gifted students in administering online peer feedback within a writing workshop for talented writers (Miller and Olthouse, 2013). The articles strive to identify the influences of using social media on students’ learning development compared to the traditional design.

### 4.2. Assessment in social media learning

A closer look at social media use in learning, assessment, and teaching reveals that various assessments have been used in different contexts. This section thoroughly analyses various social media used for assessment, the type of assessment used, and the outcome measured in the studies. The results below answer our Research Question 2: *What and how have social media been used for assessment?*

As evidenced in Table 3, various social media platforms have been identified, including Facebook (*n* = 7), WhatsApp (*n* = 3), Twitter (*n* = 1), Line (*n* = 1), Edmodo (*n* = 1), SeeSaw (*n* = 1), Ning (*n* = 1), ZhiLiao (*n* = 1), StoryBird.Com and KidsBlog (*n* = 1), and Designing for Learning and Portfolio or D4L + P (*n* = 1). These platforms are used to implement four assessment strategies.

**Table 3:** Social media used for assessment.

Type of assessment	Social media used	Description of assessment	Study
Teacher Feedback	Facebook	Immediate feedback from teacher on the posted works.	Mukhlif and Challob (2021)
		Teacher provided feedback and students were able to reread, evaluate, and revise their own and their peers’ texts.	Annamalai and Jaganathan (2017)
		Feedback was provided in the form of formative assessment aimed at contributing to the improvement of students’ learning instead of measuring what was done. The feature of online discussion supplied teachers with sufficient information for formative assessment. However, regardless of that information, the mechanism for the assessment is not clearly mentioned.	De Barros et al. (2017)
		Students were required to produce drafts of their writings based on the three topics posted on the Facebook class page wall. These drafts were then analyzed by teachers using the designated checklist.	Vikneswaran and Krish (2016)
		Students receive timely feedback both from other students and teacher. Students were also able to collect feedback from multiple sources on Facebook as conversations were not limited to only two people. Feedback on Facebook is not limited to information but may include motivation and encouragement.	Kio (2015)
	Ning	Teachers provided feedback on their published work on the blog	Casey (2013)
	An online tool, Designing for Learning and Portfolio (D4L + P),	Teachers and experts assess students’ proposal also based on the marking scheme rubric.	Wuttisela et al. (2016)
	Line App Instant Messaging and Facebook Instant Messaging	Students in the experiment class were assigned to a classroom assessment graded by teachers based on topic selection, logo structure, logotype structure, color application, and symbol pattern	Liu and Ko (2020)
	WhatsApp	Teachers delivered feedback within the process planning (class assignment). Teacher also reminded students about the deadline for projects and assignment.	Nuray (2019)
		The constant feedback exchanged between teacher and student during the ongoing work of completing assigned tasks on the Mathematical field	Bezerra et al. (2021)
		Teachers provided feedback on students’ responses and work posted on WhatsApp during the asynchronous learning.	Herwin et al. (2021)
Peer-Feedback	Facebook	Immediate feedback from other students on the posted works	Mukhlif and Challob (2021)
		Peers provided feedback so that students were able to reread, evaluate and revise their own and their peers’ texts. It motivated students to help each other;	Annamalai and Jaganathan (2017)
		Students receive timely feedback both from other students and teacher. Students were also able to collect feedback from multiple sources on Facebook as conversations were not limited to only two people.	Kio (2015)
		comments and likes from their friends on Facebook page during the writing process	Vikneswaran and Krish (2016)
	Line App Instant Messaging and Facebook Instant Messaging	Discussions and feedback among students and between the students and instructors using social media platform	Liu and Ko (2020)
	Edmodo	Students in the experimental outlined the main ideas and supporting ideas of their essay (pre-test). Then, all respondents in the group reviewed (peer-feedback) the outline. Based on those feedbacks, the students then wrote their first draft (post-test).	Yuk and Yunus (2021)
	ZhiLiao	One student answered the other student’s questions or responded to the other student’s thoughts on the platform.	Yu et al. (2020)
	SeeSaw	Student engaged and interacted around peer works. They commented on their friends’ work on SeeSaw. The peer-reviewed occurred when student commented each other’s’ work.	Lai et al. (2020)
	An online tool, Designing for Learning and Portfolio (D4L + P),	Each student gave feedback for the project proposals of the three files and assessed the project proposals based on Marking Scheme Rubric. Points for effort were awarded (1 to 5) for completion of the proposals.	Wuttisela et al. (2016)
	Ning	Quick feedback and responses on synchronous discussion. Students commented on the platform and provided constructive feedback to their peers. As part of this peer-to-peer interaction, students had three peer mentors who were asked to provide constructive feedback on the student’s work, and these mentors were eventually expected to give peer assessment.	Casey (2013)
	Social writing sites, Storybird.com and KidsBlog	Offline and online. First, Offline feedback was submitted on a teacher designed rubric, and then incorporated into a revised manuscript using Microsoft Word. Each comment was coded according to one of the levels of Bloom’s Taxonomy. Second, online feedback included students’ writings in social writing sites. Each comment was coded according to one of the levels of Bloom’s Taxonomy.	Miller and Olthouse (2013)
Pre-test and Post-test	Facebook	Pre-test was given in the pre-study phase by representing mini dialogues with omitting unneeded letters. The treatment phase was during week 2–11, and the post-test was given in week 12 by representing crossword puzzle.	Mukhlif and Challob (2021)
		Student discussed seven scientific news posted on Facebook. The questionnaires were asked before the intervention (the discussion), and immediately after. The test was administered to the students 3 weeks after the intervention (Retention Post-Test)	Huang et al. (2014)
	Twitter	Pre-test was applied to the students to examine their English level. Post-test was applied to determine whether there has been any writing skills improvement or setback. Similar tests were applied in both pre and post-test consisting of vocabulary and grammar. The topics are taken from their textbooks.	Espinoza-Celi and Pintado (2020)
	Edmodo	The writing product were evaluated for pre-test and post-test using Peer-Modo rubric (content, format and organization, and mechanics and grammar):	Yuk and Yunus (2021)
Self-Assessment	Zhi Liao	Some tools are provided on the platform, including some suggested self-evaluating learning.	Yu et al. (2020)

#### 4.2.1. Peer feedback

Peer feedback is the most common assessment strategy implemented using social media. Students engage and interact with their peers’ work posted on social media. They engage in peer feedback through reading, reviewing, and commenting on their peers’ work (Vikneswaran and Krish, 2016; Lai et al., 2020). Kio (2015) cites that using social media to elicit and provide peer feedback has benefits regarding the timeliness of the feedback received by students. In addition, students can benefit from various feedbacks received as the interactions and conversations are not limited to two individuals. However, the large number of feedback received by individual students may be overwhelming, and thus, Casey (2013) proposes an approach by assigning three peers to provide feedback to individual students. Some studies use rubrics as guidelines for peer feedback. Yuk and Yunus (2021) used the Peer-Modo rubric for the content, format, and organization, and mechanics and grammar feedback. Wuttisela et al. (2016) used rubrics to assess content and student effort in completing their work.

#### 4.2.2. Teacher feedback

In teacher feedback, there are two general approaches used. First, immediate teacher feedback is used when students publish their works on social media. Teachers provide immediate feedback upon reading and commenting on students’ posted work. This process is seen to achieve timeliness of feedback where students can immediately reflect on feedback and act on it to revise their work (Kio, 2015; Annamalai and Jaganathan, 2017; Mukhlif and Challob, 2021). In addition to commenting on students’ work, some teachers utilize this feedback approach to remind students of the deadline for uploading projects and assignments (Nuray, 2019). The feedback also serves as a mechanism for teacher to strengthen students’ understanding on the learning topic since learning does not take place in a real time context/distance learning (Herwin et al., 2021). The feedback is also viewed as a support system for students which facilitate them to reflect, analyze, and judge their own ideas when completing their assigned tasks (Bezerra et al., 2021).

Other articles report a more purposeful feedback mechanism. Using rubrics helps teachers and students improve clarity on the feedback process and content, which benefits students to improve their work further. Wuttisela et al. (2016) and Liu and Ko (2020) illustrate how teachers use rubrics in assessing students’ work and providing detailed feedback based on criteria and standards included in the rubrics. Meanwhile, Vikneswaran and Krish (2016) use a checklist to analyze the students’ writing posted on the Facebook wall. This checklist helps to ensure a more consistent and reliable assessment of and feedback on students’ work.

#### 4.2.3. Pre-test and post-test

Four articles employed social media to administer the pre/post-tests. Mukhlif and Challob (2021) and Huang et al. (2014) used Facebook to administer the pre-test and post-test. The former administered the pre-test by posting mini dialogues with omitted letters. The treatment period included weeks 2–11, and the post-test was given in the form of a crossword problem in week 12. The latter study utilized Facebook to engage students in discussing scientific news as a pre-test and post-test, which were administered before and after the intervention.

A study by Yuk and Yunus (2021) used Edmodo to administer the pre-test by providing an overview of the essay’s key points and supporting arguments to the student in the experiment group. All group respondents reviewed the outline as peer feedback. The students then composed their initial draft based on these comments, which was regarded as the post-test. Meanwhile, Espinoza-Celi and Pintado (2020) administered the pre-test and post-test consisting of vocabulary and grammar derived from the students’ textbooks *via* Twitter. Both tests were delivered to determine their initial English proficiency in writing. Another post-test was also used to examine students’ knowledge retention, where students were asked questionnaires 3 weeks after the intervention.

#### 4.2.4. Self-assessment

One article demonstrates the use of social media for self-assessment. The study of Yu et al. (2020), conducted on junior high school graduates, included students monitoring their learning using the self-evaluation checklist embedded in the platform and self-regulation through their completed learning and assessment activities.

### 4.3. Issues in using social media for assessment purposes

Three emerging issues have been identified when using social media for assessment purposes: workload issues, students’ inappropriate use of social media, and low student feedback literacy. These issues answer Research Question 3: *What are the issues with using social media in assessment?*

Apart from its benefits, five articles highlight emerging issues related to administering assessments through social media.

#### 4.3.1. Workload issues

Due to its affordances for synchronous and asynchronous engagement, teachers reported receiving messages even at unreasonable hours. They describe spending more time responding to students’ enquiries and providing feedback on their work (Nuray, 2019). The affordances of social media for flexibility blur the distinction between work and personal life. In addition, teachers report allocating extra hours to monitor students’ engagement in assessment to check possible student outputs, particularly if the outputs are publicly available (Nuray, 2019). They are forced to check students’ responses to assessment activities for language errors for fear of being criticized publicly (De Barros et al., 2017).

#### 4.3.2. Students’ inappropriate use of social media

One study raises an issue related to inappropriate language when responding to and commenting on other students’ work. In a student discussion using the WhatsApp platform, Nuray (2019) reports that some students used inappropriate and offensive language in an educational environment. Also, students tend to share unrelated and inappropriate materials embedded in their responses or feedback on social media. In both scenarios, teachers need to moderate the discussions among students. Since feedback from students is not always positive, teachers need to ensure that they thoroughly moderate peer feedback as soon as students post them (Kio, 2015).

#### 4.3.3. Insufficient feedback literacy for students

Two articles highlight the problem with students’ feedback literacy, which is the ability of the students to give feedback to their peers or to reflect and act on teacher feedback to improve their work. A study by Annamalai and Jaganathan (2017) shows that proper feedback provision would benefit students to deliver more insightful feedback to their peers’ writing. It would also leverage students’ capacity to become self-critical when performing their writing works.

Meanwhile, a study by Miller and Olthouse (2013) discusses some emerging issues with student feedback. First, vague comments on students’ feedback. Students’ opinions were frequently expressed in social media content without a clear conception of their thoughts. This attitude is known as “informal lingo,” where students provide vague comments that do not provide specific insights to help their peers improve their output. Second, informal language is used in giving feedback instead of formal language. Students assume an informal linguistic function within this social media use. Rather than replying within the formal confines of the online writing space, students write as if they were texting the writer or making a Facebook comment. In addition, there are students who are reluctant to engage in peer feedback. The reluctance to publish anything too detailed may be due to the social view that criticizing a piece of writing has a bad connotation and might influence social relations with their classmates.

### 4.4. Recommendations from the Studies

Two main recommendations emerge from previous studies to provide a better learning and assessment experience: students’ readiness to learn on social media and teachers’ preparation to learn on social media.

#### 4.4.1. Students’ readiness to use social media in assessment

A study by Miller and Olthouse (2013) recommends some critical aspects of developing student readiness. Foremost, teachers must act as role models on how students should administer good feedback that promotes students’ critical thinking. Social support for students using technology in learning is essential for effective assessment processes. A supportive learning experience will help students understand that providing constructive feedback is not always praising and identifying their peers’ strengths. Instead, it is about exercising critical thinking skills to identify areas that can further be improved and, if possible, suggest strategies to improve. In addition to those challenges, two studies highlight the importance of providing explicit training to students before engaging them in peer feedback (Miller and Olthouse, 2013; Annamalai and Jaganathan, 2017). This will address the purposes, content, process, and outcomes of engaging students in assessment.

#### 4.4.2. Teachers’ preparation to learn on social media

Two studies mention the importance of preparing teachers to administer assessment on social media. A study by Liu and Ko (2020) argues that teachers should be prepared to incorporate social media into their assessment practices. Teachers also need to be aware that students’ use of social media requires close supervision and immediate feedback. In addition, a study by Vikneswaran and Krish (2016) reinforces the argument that teachers need to explore new ways of assessing students’ learning using social media. It is inevitable that social media use for learning purposes has increased, so teachers should break away from the traditional method and embrace the contemporary methods to accommodate the needs of today’s generation of students.

## 5. Discussion

Our study provides an initial step to understanding how social media for assessment purposes has been researched and reported in the literature and identifies future directions for practice and research. There are noteworthy findings that emerged in this paper.

Foremost, there is emerging evidence that social media can be used as alternative platforms for assessment purposes. Outcomes published are mostly positive ranging from providing timely and effective feedback (e.g., Annamalai and Jaganathan, 2017; Nuray, 2019; Espinoza-Celi and Pintado, 2020; Liu and Ko, 2020; Mukhlif and Challob, 2021), monitoring student progress and providing support to those who need assistance (Espinoza-Celi and Pintado, 2020; Liu & Ko, 2020), increased student outcomes (De Barros et al., 2017; Lai et al., 2020; Yuk and Yunus, 2021), and improving teachers’ practices through the reflection of their experience (De Barros et al., 2017). The outcomes reported by the 17 studies provide preliminary evidence that social media can serve as alternative platforms for implementing various assessment strategies. Despite methodological differences among studies investigating the use of social media for assessment purposes, positive outcomes have been reported. Eliciting and giving feedback using social media has a positive impact on improving student learning (Kio, 2015; Annamalai and Jaganathan, 2017; Mukhlif and Challob, 2021). The immediate feedback received by students enables them to revise their work, which is consistent with the principle of effective feedback practices where timeliness is critical for supporting student learning (Hattie and Timperley, 2007; Black and Wiliam, 2018). Positive outcomes are also found in administering peer assessment using social media (Casey, 2013; Miller and Olthouse, 2013; Yuk and Yunus, 2021). Individual students benefit from the feedback provided by their peers. The same evidence is seen in face-to-face peer assessment activities where students’ engagement in identifying some areas needing improvement and analyzing their classmate’s work and identifying some areas needing improvement improve their learning (Hovardas et al., 2014). Students who receive peer feedback can use it to revise their work while those who give feedback benefit from understanding how their classmates interpret the assessment, which can provide them another lens to review their work (Kaufman and Schunn, 2011). Moreover, self-assessment is also successfully implemented using social media. Although the study of Yu et al. (2020) is limited only to using a checklist to engage students in self-assessment, it has demonstrated that this process increases student-regulation. The intersection between self-assessment and self-regulation was shown by Yan et al. (2020), although they used diaries to engage students in self-assessment.

There are three major issues on the use of social media for assessment purposes, including teacher working, inappropriate use of technology and student engagement and feedback literacy. Firstly, the workload implications of using social media. Due to the accessibility and affordances of social media for immediate feedback (Alenezi and Brinthaupt, 2022), teachers spend more time reading students’ work and providing feedback (Nuray, 2019). Teacher workload in assessment has been an ongoing issue especially when providing individual feedback for many classes (Carless and Winstone, 2020), but there is extra pressure in an online platform because it is publicly available. Teachers take extra time to correct students’ work for fear that the output of students will reflect the effectiveness of their teaching (De Barros et al., 2017). Secondly, there is a tendency for students to use inappropriate language when responding to and commenting on their peer’s work (Nuray, 2019). Addressing this concern requires enhancing students’ digital literacy to become responsible users of social media to support their learning without the risk of engaging in inappropriate behavior online (Gleason and Sam von, 2018). Thirdly, the effectiveness of using social media for assessment purposes depends largely on students’ effective engagement in assessment (Miller and Olthouse, 2013; Annamalai and Jaganathan, 2017). This means that students should have a certain level of assessment knowledge and skills, known as assessment literacy (Hannigan et al., 2022), to understand the purpose and aim of assessment, and the processes required to optimize the impact of their engagement in assessment on their learning. In our findings, students’ ability to engage in peer assessment is limited by their insufficient feedback literacy. There are students who provide vague comments that are not aligned to the learning outcomes and criteria, which the recipient finds difficult to act on (Miller and Olthouse, 2013).

Apart from the outcomes and issues reported, there are significant gaps we have identified. With only 17 relevant articles extracted from three databases, this number indicates that research on the use of social media for assessment purposes is very limited. Among those studies, 66% were conducted in Asian countries, with only one study from countries like the United States, Australia, Brazil, Ecuador, and Turkey. The limited number of studies highlights one apparent gap in the literature in terms of the adoption of social media for assessment purposes. We cannot make any assumptions relating to the preferential use of social media or issues that make them less popular for assessment purposes. In addition, it is difficult to make inferences about the contextual differences in using social media for assessment purposes, even though there is a range of evidence for the context-based nature of assessment (Taylor, 2013; Alonzo et al., 2021). Notably, 35.39% of the studies synthesized focus on language learning, and thus limit the insights of the utility of social media in other key learning areas. The use of social media in other key learning areas is worthwhile exploring because the effectiveness of assessment requires adaptation for each key learning area (Scarino, 2017). What is evident at this stage is that critical enquiries about the intersections of assessment and social media are limited only to exploring if they can be used as alternative platforms.

Moreover, in 52.94% of the studies, the use of social media for assessment purposes is tacked on to the pedagogical use of assessment. This approach is consistent with the true nature of the intersections of assessment, learning, and teaching, where assessment is the central feature of learning and teaching (Broadfoot et al., 1999). Investigation of assessment within a broader pedagogical model is ideal because it highlights how assessment is used to inform learning and teaching activities (Black and Wiliam, 2018). However, in the studies mentioned above, the link between assessment, learning and teaching and their intersections with the use of social media are not explicitly discussed. In most articles (76.47%), assessment is not clearly positioned as driving the learning and teaching activities. The disconnect between assessment and pedagogy is one of the major criticisms in assessment (Baird et al., 2017). For research involving the pedagogical functions of assessment, the findings must clearly demonstrate how assessment and assessment data inform pedagogical approaches, such that learning, and teaching activities are modified to adequately address the learning needs of the students (Alonzo, 2016, 2020).

In addition, research on the use of social media for assessment is fragmented. There is a distinct gap in the literature regarding the effective use of social media for assessment purposes. The studies analyzed in this paper did not explicitly investigate the intersections between assessment and social media. At this stage of research, what has been reported is the outcomes of using social media and the gains in student outcomes. We need broader investigations of the direct and indirect effects of using social media for assessment on teacher pedagogical approaches and student learning, engagement, and outcomes. Additional investigations should critically explore what factors influence teachers and students to use social media for assessment purposes. The question of whether factors of convenience or affordances influence the use of specific social media remains inconclusive.

Even more so, the number of social media reports for assessment purposes is limited. Only nine social media have been reported, with Facebook being the most popular (e.g., Huang et al., 2014; Annamalai and Jaganathan, 2017; De Barros et al., 2017). This limited number of research presents an enormous opportunity as most of the students and teachers are using or have access to social media. Exploring other social media platforms for assessment purposes may highlight two important issues: What should be the affordances of social media to become effective assessment platforms and are social media perceived by teachers and students to be just for social networking?

Subsequently, another significant gap is the investigation of teachers’ and students’ dispositions in using social media for assessment purposes. It is well documented that a person’s beliefs and perceptions influence their actual action (Fives and Buehl, 2016; Beswick and Alonzo, 2022); hence, this critical enquiry area is notably important to contribute to the theorisation of using social media for assessment purposes. The interplay between teachers’ and students’ dispositions, knowledge and skills will provide insights into social media adoption in the assessment context. This gap in the literature is directly related to the issue raised above on the perception of teachers and students on the function of social media.

Finally, the discussion around ensuring the integrity of assessment implemented using social media is lacking in the literature. Issues related to trustworthiness (Alonzo, 2020), ethical practice and cheating (Sadler, 2009) in online assessments continue to raise validity concerns.

## 6. Conclusion

Our study aimed to investigate the extant literature on how social media are used for assessment purposes. Our review of the literature using the PRISMA methodology extracted 17 articles from three databases. Our findings indicate emerging evidence that social media can be used as alternative platforms to implement assessment strategies. There are reported positive outcomes, including providing timely and effective feedback, monitoring student progress and providing support to those who need assistance, increasing student outcomes, and improving teachers’ practices. There are also issues that hinder the effectiveness of social media for implementing assessment. These include increased teacher workload, the need for constraint moderation of students’ peer feedback, particularly if they are publicly available, and students’ feedback literacy. We need more studies to better understand the intersections of social media, assessment and learning to develop a strong theorisation.

Like many studies, we acnowledge some limitations to our research. The combination of our keywords might have limited our search. For future studies, the social media keyword can be further expounded to include all other emerging social media platforms. Also, we excluded gray literature, book chapters, reports, and books. These publications might have other critical information about using social media for the implementation of assessment. Furthermore, we did not include studies from the higher education context. It would be worthwhile to explore how higher education uses social media for assessment purposes.

## Author contributions

DA conceptualized the paper, identified the key insights, searched the literature, performed coding and analysis, wrote the draft, and monitored the progress of the team. CO conceptualized the paper, searched the literature, performed coding and analysis, and wrote the draft. WW performed coding and analysis and wrote the draft. CH edited and proofread the paper and wrote the conclusion section. All authors contributed to the article and approved the submitted version.

## Funding

We would like to acknowledge the financial support under the 2021 Faculty of Arts, Design and Architecture (ADA Research Fellow Scheme).

## Conflict of interest

The authors declare that the research was conducted in the absence of any commercial or financial relationships that could be construed as a potential conflict of interest.

## Publisher’s note

All claims expressed in this article are solely those of the authors and do not necessarily represent those of their affiliated organizations, or those of the publisher, the editors and the reviewers. Any product that may be evaluated in this article, or claim that may be made by its manufacturer, is not guaranteed or endorsed by the publisher.
